# *Lavandula dentata* leaves as potential natural antibiofilm agents against *Pseudomonas aeruginosa*

**DOI:** 10.1038/s41598-025-88824-5

**Published:** 2025-03-12

**Authors:** Maram M. Aboulwafa, Nada M. Mostafa, Fadia S. Youssef, Omayma A. Eldahshan, Abdel Nasser B. Singab

**Affiliations:** 1 Faculty of Pharmacy, Department of Pharmacognosy, Ain Shams University, Abbassia, 11566 Cairo Egypt; 2 Center of Drug Discovery Research and Development, Faculty of Pharmacy, Ain Shams University, Abbassia, 11566 Cairo Egypt

**Keywords:** Skin, Burn, Biofilm, *Lavandula dentata*, *Pseudomonas aeruginosa*, Sagerinic acid, Biofilms, Bacterial infection, Secondary metabolism, Drug discovery and development, Medical research

## Abstract

Biofilm formation is responsible for persistent diseases related to chronic infections. Mostly it is triggered by many bacteria, mainly *Pseudomonas aeruginosa* (*P. aeruginosa*). In this study, plants that have been used traditionally in skin infections Viz; Liquorice, Carrot, Red Cabbage, Beetroot, Turmeric, Neem, and French Lavender were selected to evaluate their antibiofilm activity against *P. aeruginosa*. The microtiter plate assay was used to evaluate their antibiofilm activity against *P. aeruginosa* as well as ability to reduce the activity of *P. aeruginosa*. To investigate the phytocompounds responsible for bioactivity of the superior extract and to explore potential interactions between its bioactive components and one of quorum-sensing (QS) regulatory proteins of *P. aeruginosa* involved in biofilm formation, liquid chromatography-mass spectrometric (LC-MS) and molecular docking studies were done. The study showed that all tested plant extracts could significantly (*p*-value < 0.05) reduce the formation of *P. aeruginosa* biofilm. The methanol extract of *Lavandula dentata* (*L. dentata*) leaves is superior at 0.625 mg/mL. In conclusion, the study revealed the presence of phenolic acids, flavonoids, and their glycosides also, the anti-*P. aeruginosa* biofilm activity of *L. dentata* leaves was reported herein for the first time and could be a good source of leads for antibiofilm medicine.

## Introduction

Biofilms can profoundly impact human health. In medical contexts, biofilms present a significant challenge as they facilitate microbial persistence and resistance to antibiotics, leading to chronic infections. Biofilm is defined as: “A structured community of bacterial cells enclosed in a self-produced polymeric matrix and adherent to an inert or living surface”. These resilient structures can form on various surfaces within the body, such as catheters, implants, and tissues, exacerbating conditions like urinary tract infections, periodontal disease, respiratory infections, and chronic wounds. Chronic wounds were reported to be an ideal environment for biofilm formation which is a critical element in certain skin diseases. Burn injury, if improperly treated, will be an ideal environment for biofilm formation triggered by many bacteria. Bacterial infection shows signs of a crucial role in morbidity and mortality of patients with burn wounds/infections^1^.

*P. aeruginosa* is one of the common pathogens causing refractory infection in burn patients using deep implanted vein catheters^2^. *P. aeruginosa* is a good biofilm producer that aggravates the condition of burns patients by being slow or non-responsive to antibiotics^3^. *P. aeruginosa* has acquired resistance to commonly used antibiotics and is now a priority pathogen on the Centers for Disease Control and Prevention ESKAPE pathogen list. Developing strategies to disrupt or prevent biofilm development is crucial for combating biofilm-associated health complications and improving patient outcomes. The use of antibiofilm agents is a novel strategy for the prevention and treatment of infections of burn wounds.

Natural products were widely employed for many centuries as raw materials for the manufacturing of various pharmaceuticals, cosmeceuticals, and nutraceuticals^4,5^. Nowadays, traditional medicines are gaining a lot of attention in global healthcare considerations. Besides, they are regarded as the centerpiece of about 75–80% of the world population’s healthcare, mainly in developing countries, because of their better cultural acceptability, better suitability to the human body, and lesser side effects. Skin diseases are very common in rural areas, in the developing world, where human diseases are treated with medicinal plants. Accumulated evidence demonstrated that natural products from plants had properties in the modulation of biofilm formation in the last two decades; as plant extracts showed target sites other than those used by antibiotics and hence proved their ability in the modulation of biofilm formation. The extensive literature survey revealed the idea that more than 300 plant species are used in the treatment of skin diseases under various community practices^6^.

Recognizing the prevalence of biofilm-associated skin infections and the need for novel therapeutic interventions, current investigation focused on evaluating the antibiofilm potential of seven botanical extracts traditionally utilized in treating skin diseases. Among the tested botanical extracts, one emerged as notably superior in inhibiting biofilm formation by *P. aeruginosa* which is methanol extract of *L. dentata* leaves. Encouraged by this promising finding, further investigations were initiated utilizing advanced analytical techniques such as LC-MS and molecular docking studies. These complementary approaches were employed to elucidate the chemical composition of the methanol extract of *L. dentata* leaves and to explore potential interactions between its bioactive components and LasR, QS regulatory protein^7^ of *P. aeruginosa*, a key molecular target involved in biofilm formation.

## Results

### Evaluation of the antimicrobial activity of methanol extract of the seven plants against ***P. aeruginosa***

Regarding minimal inhibitory concentrations (MIC) of plant extracts against *P. aeruginosa*, it was observed that growth occurs in all wells containing tested concentrations, and hence MICs of all tested plants along with acetylcysteine (ACC) (reference compound) against *P. aeruginosa* were greater than 5 mg/mL since it was reported that when growth occurs in all dilutions containing the antimicrobial agent, the MIC is recorded as greater than the highest concentration^8^.

### Evaluation of the antibiofilm activity of methanol extract of the seven plants

**Fig. 1:**
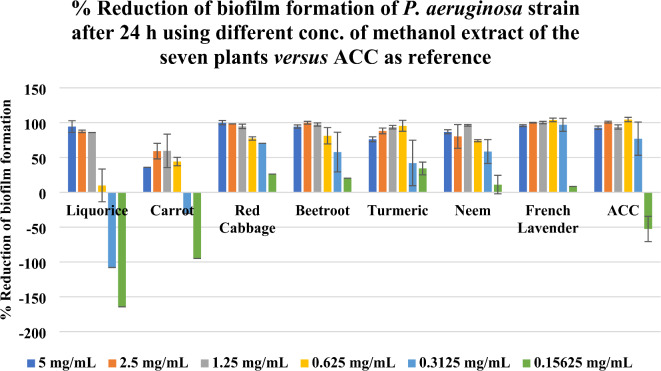
%Reduction of biofilm formation of *P. aeruginosa* strain after 24 h using different conc. of methanol extract of the seven plants *versus* ACC as reference (Each column is represented as mean ± standard error).

**Fig. 2:**
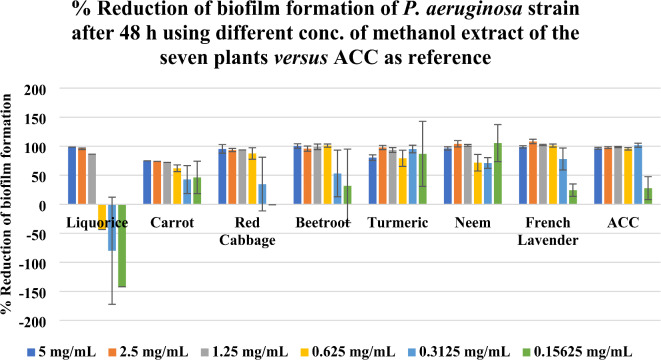
%Reduction of biofilm formation of *P. aeruginosa* strain after 48 h using different conc. of methanol extract of the seven plants *versus* ACC as reference (Each column is represented as mean ± standard error).

Percentage (%) reduction of biofilm formation of *P. aeruginosa* strain using different concentrations of methanol extract of the seven plants *versus* ACC as reference was illustrated in Figs. 1 and 2 where the obtained findings were represented as clustered columns charts as shown in Fig. 1 for the 24 h incubation period whereas Fig. 2 displayed the 48 h incubation period.

It was observed that French lavender, neem, turmeric, and beetroot have a high % reduction of biofilm formation in both 24 h and 48 h incubation periods, compared to ACC and in all their used concentrations. Red cabbage, as well, has a high % reduction of biofilm formation along its all-used concentration in case of 24 h incubation period, but it loses its activity at its lowest concentration (0.15625 mg/mL) in case of 48 h incubation period. However, carrot has a high % reduction of biofilm formation along its all used concentration, except the two lowest ones (0.3125 mg/mL and 0.15625 mg/mL) in case of 24 h incubation period, but it regains its activity in case of 48 h incubation period. Regarding liquorice, it has a % reduction of biofilm formation, especially at concentrations 5 mg/mL, 2.5 mg/mL, and 1.25 mg/mL. It was noted that % reduction may appear in a negative sign in the case of low concentration of liquorice extract and the case of ACC as well. Through comparing those seven plant extracts at each used concentration, results showed that methanol extract of *L. dentata* leaves displayed the most superior activity among the seven tested plant extracts with % reduction of biofilm formation ranges from 8.5 to 104.0% ± 2.7% in case of 24 h incubation period, and from 24.4 to 108.2% ± 3.9% in case of 48 h incubation period and with a concentration of 0.625 mg/mL being the optimum one for its activity; as it was the concentration at which methanol extract of French lavender functioned at its best in case of 24 h incubation period and below it, the activity began to decline in case of 48 h incubation period.

### Statistical analysis of the results of % reduction of biofilm formation of methanol extract of the seven plants

In the case of the 24-hour incubation period, the two-way analysis of variance (ANOVA) revealed that F (4.19) > F_critical_ (2.11) for factor 1 (type of extract) at *p*-value 0.00046 (< 0.001). The mean of French lavender extract (82.12%) is the highest among the seven plants and even higher than that of ACC (47.76%). There was also a significant main effect of concentration (factor 2), F (23.68) > F_critical_ (2.309) at *p*-value 1.20 × 10^− 15^ (< 0.001). The interaction between extract type and concentration was significant, F (1.93) > F_critical_ (1.55) at *p*-value 0.0062 (< 0.01). Otherwise, in the case of the 48 incubation period, F (3.67) > F_critical_ (2.11) for factor 1 (type of extract) at *p*-value 0.0015 (< 0.01). There was also a significant main effect of concentration (factor 2), F (5.04) > F_critical_ (2.31) at *p*-value 0.00038 (< 0.001). The interaction between extract type and concentration was significant, F (1.61) > F_critical_ (1.55) at a *p*-value of 0.035 (< 0.05).

### Chemometric analysis of the % reduction of biofilm formation of methanol extract of the seven plants

**Fig. 3:**
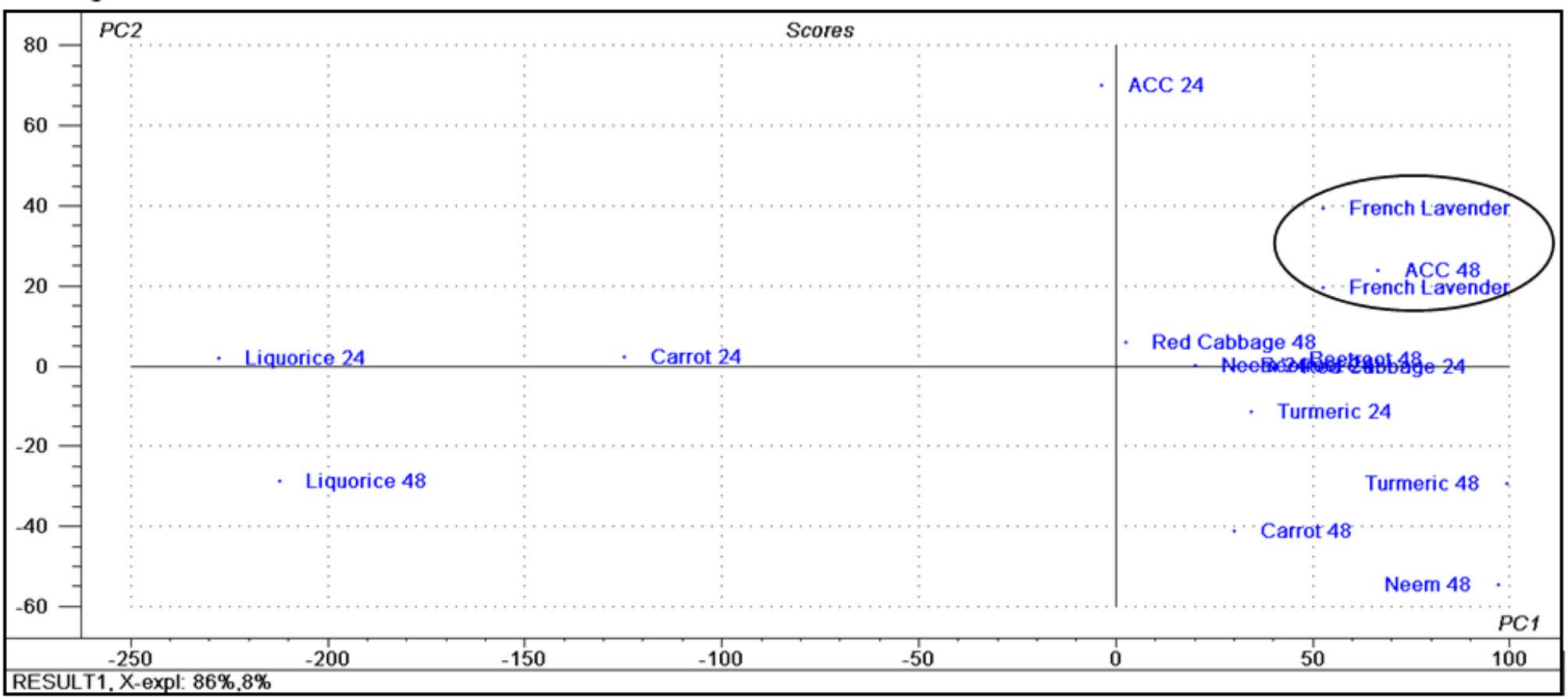
PCA score plot of the % reduction of biofilm formation data matrix of the methanol extract of the seven plants after both 24 h and 48 h incubation periods.

**Fig. 4:**
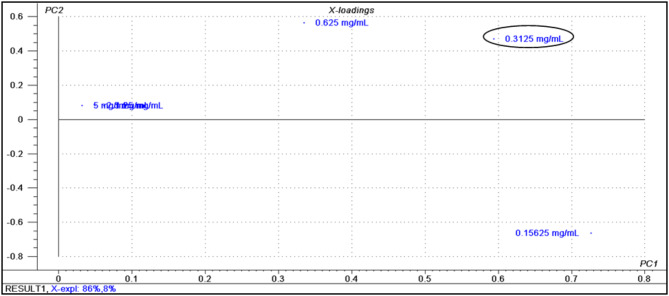
Loading plot of the % reduction of biofilm formation data matrix of the methanol extract of the seven plants after both 24 h and 48 h incubation periods.

The principal component analysis (PCA) score plot resulted in two orthogonal PCs, which explained 94% of the variance using only the first two components (the first PC accounts for 86% of the total variance followed by the second PC with 8%). The PCA score plot of the % reduction of biofilm formation data matrix of the methanol extracts of the seven plant samples after both 24 h and 48 h incubation periods was displayed in Fig. 3. By careful inspection of the plot, the results confirmed the closeness of French lavender in its activity against biofilm formation by *P. aeruginosa* to the reference used (ACC). The loading plot displayed in Fig. 4 showed that 0.3125 mg/mL concentration was able to differentiate between the used plant extracts regarding their activity. This observation explains the clustering of carrot extract after 48 h incubation period on the right half of the plot concerning PC1, which gains its activity at 0.3125 mg/mL after 48 h incubation period and correlates with our findings that liquorice has no activity against biofilm formation at concentration 0.3125 mg/mL; as it is clustered on the left half of the plot concerning PC1 in case of both 24 h and 48 h incubation periods; owing to concentrations 5 mg/mL, 2.5 mg/mL and 1.25 mg/mL as shown in Fig. 4.

### Chemical investigation of methanol extract of *L. dentata* leaves using LC-MS analysis

**Fig. 5:**
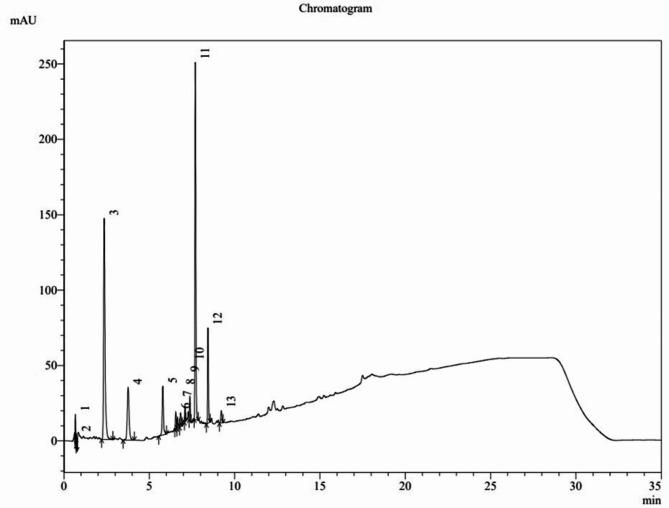
The UPLC chromatogram of methanol extract of *L. dentata* leaves at λ_254 nm_.

**Fig. 6:**
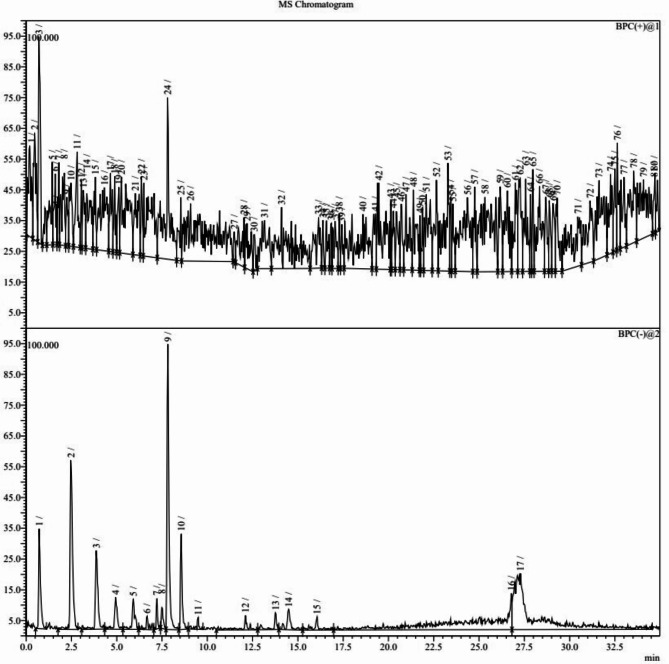
The mass chromatogram in positive ion mode (upper chromatogram) and negative ion mode (lower chromatogram) of methanol extract of *L. dentata* leaves.

**Fig. 7:**
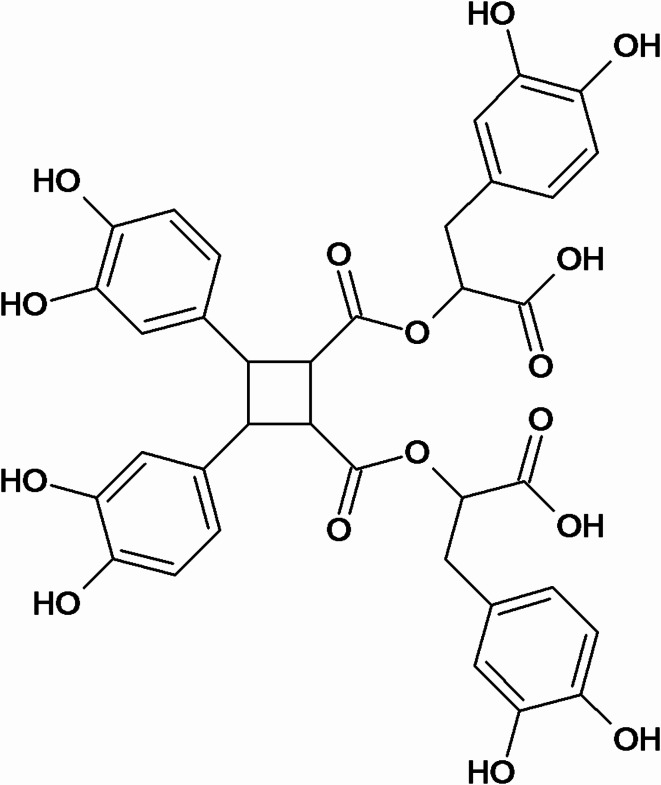
Chemical Structure of Sagerinic acid.

The ultra-performance liquid chromatography (UPLC) chromatogram of methanol extract of *L. dentata* leaves at wavelength (λ) 254 nm is shown in Fig. 5. The mass chromatograms of methanol extract of *L. dentata* leaves in positive and negative modes, showing eighty one and seventeen peaks respectively, are shown in Fig. 6. Thirty-two compounds were tentatively identified by LC-MS from methanol extract of *L. dentata* leaves as shown in Table 1. A dimer of rosmarinic acid, which is identified as sagerinic acid^9^, was found to be the major identified compound accounting for 10.38% with a chemical structure as given in Fig. 7 whereas other hydroxycinnamic acids include caffeic acid, coumaric acid, ferulic acid, caffeoylquinic acid (chlorogenic acid, cryptochlorogenic acid or neochlorogenic acid), fertaric acid and chicoric acid. Seven compounds belonging to hydroxycinnamic acid derivatives and three compounds belonging to benzoic acid derivatives were identified in addition to thirteen compounds belong to flavonoids.

**Table 1:** List of the tentatively identified compounds from methanol extract of *L. dentata* leaves along with the peak number (No.) at which this compound was isolated, retention time (t_R_), mass-to-charge ratio (*m/z*) and the detected molecular weight (g/mol).

No	t_R_ (min)	Area %	*m/z*		M. wt (g/mol)	Identified compounds	References
			[M + H]^+^	[M-H]^-^			
1	0.19	0.39	183		182	Dihydrocaffeic acid (1)	^10^
2	0.47	0.37	200		199	Not identified	
3	0.71	0.51	381		380	4-Hydroxybenzoic acid 4-(6-*O*-sulfo) hexoside	^10^
			247		246	N-Acetyltryptophan	^11^
4	1.05	0.10	183		182	Dihydrocaffeic acid (2)	^10^
5	1.44	0.25	183		182	Dihydrocaffeic acid (3)	^10^
			329		328	Homovanillic acid rhamnoside (1)	^12^
6	1.6	0.18	224		223	Not identified	
7	1.81	0.12	411		410	Vanillic acid sulfoquinovoside	^10^
			183		182	Dihydrocaffeic acid (4)	^10^
8	2.1	0.31	185		184	Methyl gallic acid derivative	^13^
9	2.24	0.12	1456		1455	Not identified	
10	2.48	0.23	165		164	Not identified	
11	2.81	0.30	367		366	Not identified	
12	3.05	0.08	355		354	Chlorogenic acid or Cryptochlorogenic acid or Neochlorogenic acid	^10,14^
13	3.16	0.18	767		766	Not identified	
14	3.36	0.33	181		180	Caffeic acid	^15^
15	3.82	0.17	479		478	Hypolaetin 8-*O*-hexuronide	^10^
16	4.32	0.56	542		541	Not identified	
17	4.65	0.22	402		401	Not identified	
18	4.89	0.15	426		425	Not identified	
19	5.12	0.15	310		309	Not identified	
20	5.25	0.70	260		259	Not identified	
21	6.03	0.35	475		474	Chicoric acid	^10,12^
22	6.37	0.15	341		340	Esculetin-*O*-hexoside	^12^
23	6.50	0.67	285		284	Acacetin	^11^
24	7.82	0.88	383		382	Not identified	
25	8.54	0.16	1230		1229	Not identified	
26	9.07	1.61	1086		1085	Not identified	
27	11.49	0.06	1391		1390	Not identified	
28	12.03	0.25	1374		1373	Not identified	
29	12.19	0.24	477		476	Methylluteolin-*O*-hexuronide	^16^
			539		538	Lithospermic acid A	^16^
30	12.58	0.09	553		552	Methyl melitrate/lithospermate	^10^
31	13.15	0.42	961		960	Not identified	
32	14.10	0.96	607		606	Not identified	
33	16.14	0.33	1189		1188	Not identified	
34	16.38	0.07	273		272	Naringenin (1)	^15^
35	16.58	0.14	289		288	Eriodictyol	^9^
36	16.83	0.07	595		594	Kaempferol-*O*-pentose-*O*-hexuronic acid	^13^
			471		470	Umbelliferone deoxyhexosyl hexoside	^12^
37	17.06	0.19	1122		1121	Not identified	
38	17.29	0.08	341		340	Salvianolic acid G	^12^
39	17.42	0.09	1416		1415	Not identified	
40	18.59	0.80	1350		1349	Not identified	
41	19.29	0.21	1200		1199	Not identified	
42	19.46	0.62	1412		1411	Not identified	
43	20.14	0.06	1346		1345	Not identified	
44	20.27	0.16	1252		1251	Not identified	
45	20.44	0.20	1411		1410	Not identified	
46	20.71	0.17	273		272	Naringenin (2)	^15^
47	20.97	0.32	1268		1267	Not identified	
48	21.37	0.33	1148		1147	Not identified	
49	21.74	0.05	1398		1397	Not identified	
50	21.88	0.11	653		652	Tricin-*O*-feruloyl deoxyhexoside or Quercetin acetyl disaccharides	^11,13^
51	22.08	0.46	1193		1192	Not identified	
52	22.65	0.32	759		758	Not identified	
53	23.29	0.60	465		464	Quercetin hexoside	^17^
54	23.44	0.12	1017		1016	Not identified	
55	23.55	0.15	1049		1048	Not identified	
56	24.36	0.87	826		825	Not identified	
57	24.75	0.22	271		270	Apigenin	^10,11,13,15,18^
58	25.33	1.14	770		769	Not identified	
59	26.17	0.30	815		814	Not identified	
60	26.56	0.48	961		960	Not identified	
61	27.01	0.35	331		330	Pimarane diterpene (1)	^12^
			595		594	Kaempferol-*O*-deoxyhexosyl hexoside	^13^
62	27.26	0.39	960		959	Not identified	
63	27.57	0.34	331		330	Pimarane diterpene (2)	^12^
			519		518	Dihydroxy monomethoxy ursolic acid (Ursolic acid derivative)	^12^
64	27.84	0.09	489		488	Dihydroxy ursolic acid	^12^
65	27.97	0.15	895		894	Not identified	
66	28.34	0.51	960		959	Not identified	
67	28.68	0.22	505		504	Quercetin derivative	^12^
68	28.89	0.16	931		930	Not identified	
69	29.09	0.21	1170		1169	Not identified	
70	29.31	0.28	493		492	Salvianolic acid C or its isomer	^10^
71	30.46	0.64	953		952	Not identified	
72	31.13	0.47	172		171	Not identified	
73	31.62	0.68	341		340	Not identified	
74	32.26	0.33	270		269	Not identified	
75	32.49	0.21	633		632	Not identified	
76	32.63	0.24	1188		1187	Not identified	
77	33.00	0.28	247		246	Not identified	
78	33.54	0.52	429		428	Not identified	
79	34.07	0.67	957		956	Not identified	
80	34.70	0.13	288		287	Not identified	
81	34.84	0.11	278		277	Not identified	
82	0.73	3.47		387	388	Dihydrosinapic acid hexoside	^11^
83	2.47	7.98		325	326	Coumaric acid hexoside	^10,19^
84	3.88	4.01		711	712	Ferulic acid hexoside dimer	^17^
85	4.94	1.70		327	328	Homovanillic acid rhamnoside (2)	^12^
86	5.91	1.61		325	326	Fertaric acid	^10,19^
87	6.67	0.74		355	356	Ferulic acid hexoside	^10^
88	7.21	0.81		521	522	Rosmarinic acid hexoside	^10^
89	7.50	0.79		431	432	Apigenin-*O*-hexoside (Vitexin)	^18^
90	7.82	10.38		359	360	Rosmarinic acid	^11,15,17,18,19^
				719	720	Sagerinic acid	^10^
91	8.56	3.03		593	594	Not identified	
92	9.49	0.55		163	164	Coumaric acid	^15,20^
93	12.11	1.09		283	284	Not identified	
94	13.76	0.94		433	434	Quercetin-*O*-pentoside	^13^
95	14.49	1.43		327	328	Not identified	
96	16.05	1.02		1144	1145	Not identified	
97	26.78	15.15		325	326	Coumarin (1)	^21^
98	27.29	18.47		145	146	Coumarin (2)	^21^

### Docking of the identified compounds of methanol extract of *L. dentata* leaves

**Fig. 8:**
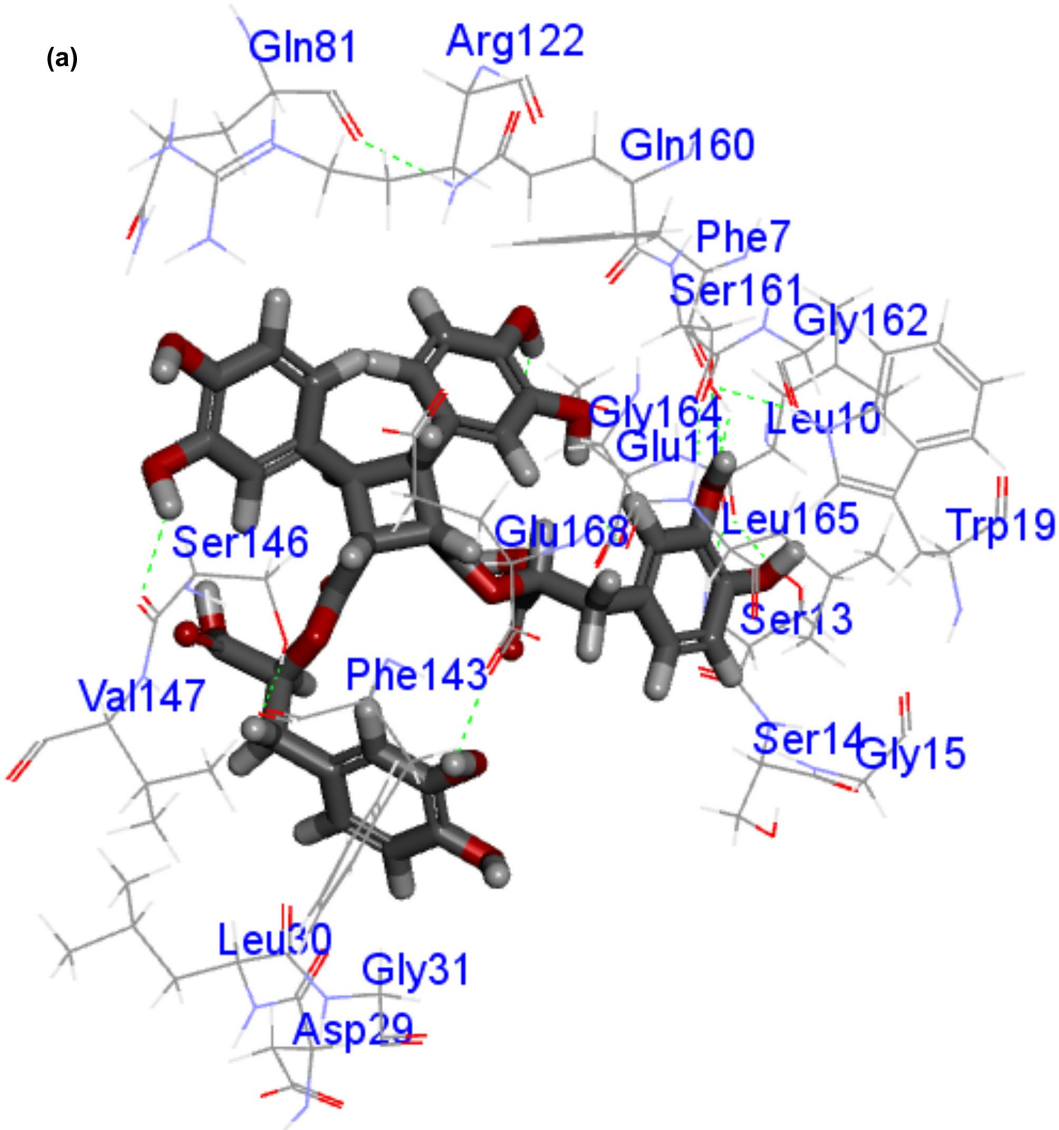

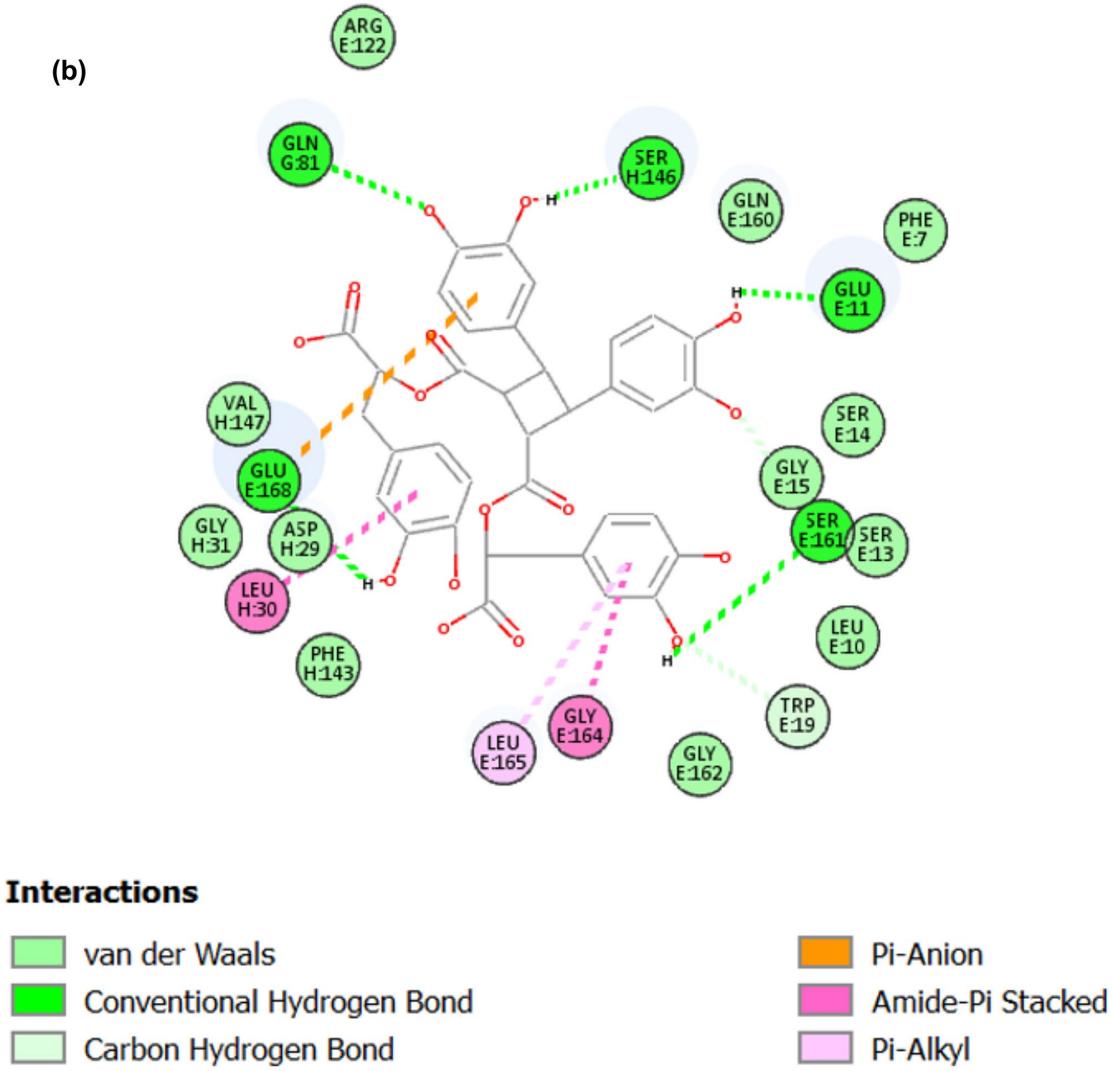
(**A**) 3D diagram of interactions of Sagerinic acid into the active site of LasR (Libdock score: 170.824). (**B**) 2D diagram of interactions of Sagerinic acid into the active site of LasR (Libdock score: 170.824).

All tested compounds were successfully docked into the active site of LasR, the Libdock scores are shown in Table 2. The highest Libdock score was 170.824 and corresponded to sagerinic acid. 3D and 2D diagrams of the highest Libdock scores docked compound which is sagerinic acid are shown in Fig. 8.

**Table 2:** List of the tentatively identified compounds from methanol extract of *L. dentata* leaves along with their calculated docking scores of the targeted LasR active site.

Identified compounds	LibDock score
Sagerinic acid	170.824
Rosmarinic acid	162.511
Salvianolic acid C	148.746
Chicoric acid	148.735
Chlorogenic acid	147.143
Umbelliferone deoxyhexosyl hexoside	145.064
Lithospermic acid A	143.462
Cryptochlorogenic acid	143.001
Neochlorogenic acid	142.14
Coumaric acid hexoside	139.557
Fertaric acid	131.583
Eriodictyol	131.488
Apigenin	128.325
Naringenin	124.533
Salvianolic acid G	124.309
Acacetin	123.279
Vitexin	121.54
N-acetyl-tryptophan	109.522
Hypolaetin-8-*O*-hexuronide	97.5477
Dihydrocaffeic acid	96.284
Coumaric acid	93.4804
Coumarin	83.3097
Acetylcysteine (Reference compound)	75.1281

## Discussion

The seven plants were selected due to their reported usage against various skin ailments^22–28^. Liquorice, red cabbage, beetroot, turmeric and neem were reported to contain compounds that disrupt the ability of *P. aeruginosa* to form biofilms^29–33^. However, the methanol extract of turmeric, in our study, achieved a higher % of reduction of biofilm formation than those achieved before by pure curcumin, the bioactive compound of turmeric^26^ indicating that the synergistic action of all the bioactive compounds discloses the antibiofilm activity of turmeric^34^. In addition, methanol extract of neem leaves achieved, in our study, a reduction of biofilm formation at lower concentrations than those reported in previous studies which may be due to the difference in the geographical location and other chemical and physical parts of the environment which affects the phytoconstituents of polar extract of neem leaves which is rich in phenolics compounds that are responsible for the different biological activities of neem^35^.

The phenomenon of the negative sign which appeared in the % reduction in the case of low used concentration of liquorice extract and in case of ACC as well is called “hormetic response”. The term hormesis, derived from the Greek “to excite,” was first proposed in 1943 by Southam and Erhlich. Hormesis is a biphasic dose-response relationship characterized by low-dose stimulation and high-dose inhibition^36^ in living organisms, which may be time-dependent. In the case of liquorice, for example, the hormesis phenomenon occurs and the stimulation is enhanced with time.

Carrot has not been studied yet for their specific effects on *P. aeruginosa* biofilm but was known for its various bioactive compounds which contribute to its antimicrobial properties. The ability of carrot extract to reduce the biofilm formation by *P. aeruginosa* is first reported here in current study. Regarding *L. dentata*, previous research on it was not as widespread as that on other *Lavandula* species. *L. dentata* is a plant commonly used in traditional medicine in various regions of Morocco and Brazil to treat a range of skin ailments^28,37^. *L. dentata* leaves were selected for this study to justify its traditional uses in skin infections, dermatological diseases, and skin burns^28,37–39^. In addition, research on its leaves was not as extensive as that on its flowering tops or essential oil. Previous studies on *L. dentata* leaves mainly investigated their essential oil composition. *L. dentata* leaves were reported herein for the first time to have anti-bacterial biofilm activity and specifically anti-*P. aeruginosa* biofilm activity.

The two-way ANOVA revealed that the % of biofilm inhibition varies based on the type of extract and differs significantly between these types of extracts. Different concentration levels had a significant impact on the % reduction of biofilm formation as well. Furthermore, the effect of concentration on the % reduction of biofilm formation varied depending on the type of extract. Methanol extract of *L. dentata* leaves exhibited a high % reduction of biofilm formation in both 24 h and 48 h incubation periods, compared to ACC, a well-known preventer of biofilm attachment. Chemometric analysis confirmed these observations as well. From this finding, the foregoing study aimed to get the benefit of the effectiveness of the leaves which represents a great point from an economic point of view. This study is among the few studies performed on *L. dentata* extract and not the essential oil. Hence, *L. dentata* leaves may not be as economically significant as other parts of the plant, but they still hold value and can be repurposed for drug discovery rather than considered waste.

Regarding the tentatively identified compounds from *L. dentata* extract, the major identified compound was sagerinic acid (rosmarinic acid dimer) which was previously identified in *L. dentata*^10^. Rosmarinic acid is the most dominantly present popular hydroxycinnamic acid of the *Lavandula* genus^40^ and was reported in hydroalcoholic extract of *L. dentata* aerial parts^19^. Other hydroxycinnamic acids such as coumaric acid, ferulic acid, fertaric acid and chicoric acid, it was the first time to be reported in *L. dentata* to the best of the authors’ knowledge.

Hydroxycinnamic acid derivatives such as dihydrocaffeic acid was reported before in *L. dentata*, but in a lower abundance than in our study^10^. Caffeic acid tetramer and trimers were also identified such as lithospermic acid A (trimer). Salvianolic acids C and C isomer (trimers) were found in *L. dentata* but in a lower abundance^10^. Rosmarinic acid hexoside was found before in *L. dentata* but in a lower abundance than ferulic acid hexoside^10^ in contrast to our study.

Regarding docking study of the identified compounds from *L. dentata* extract, it was found that all tested compounds have successfully docked into the active site of LasR and hence can act against *P. aeruginosa* virulence. Sagerinic acid (rosmarinic acid dimer) which was the most abundant compound in the LC-MS chromatogram of *L. dentata* extract accounting for 10.38%, achieved the highest Libdock score which was 170.824, which means that it could be responsible for the activity of *L. dentata* extract against *P. aeruginosa* biofilm formation. Rosmarinic acid which was reported to specifically bind to the *P. aeruginosa* RhlR QS receptor^41^, was successfully docked into the active site of LasR in the current study with Libdock score of 162.511.

Other compounds showed high Libdock scores and hence high binding affinity to QS associated LasR, which plays a pivotal role in the activation of many virulence genes in *P. aeruginosa*. Coumaric acid was previously identified as anti-*P. aeruginosa* biofilm compound. Ferulic acid was reported to reduce the production of pyocyanin (QS regulated virulence factor) that contributes to tissue damage and inflammation in case of skin infection and negatively affects various systems including the urinary system^42^. Chlorogenic acid was reported to be a QS inhibitor and inhibit biofilm formation of *Pseudomonas*. It interferes with the signaling molecule synthesis and transcription regulators using the Las, Pqs and Rhl systems^43^. Vitexin has a marked attenuation in biofilm formation and QS-mediated phenotype of *P. aeruginosa*; as it was reported to show high binding affinity with QS associated LuxR, LasA and LasI using molecular docking^44^.

The relationship between both in vitro and molecular docking studies is crucial; between studying the antibiofilm activity using a microtiter plate assay, a widely employed method to assess the ability of plant extracts to disrupt biofilm formation in *P. aeruginosa* in vitro and molecular docking study against LasR, QS regulatory protein of *P. aeruginosa*. LasR controls the expression of genes involved in biofilm formation, including those responsible for the production of extracellular polymeric substances that are essential for biofilm stability, hence compounds that inhibit LasR activity can prevent or reduce biofilm formation by interfering with quorum sensing signals, thus reducing the expression of LasR-regulated genes. By using the microtiter plate assay, we hence evaluated how potential LasR inhibitors impact biofilm formation and determined their antibiofilm efficacy, which is an important step in developing treatments for chronic infections associated with biofilms.

Plant extracts showed antibiofilm activities; due to the synergistic activities of their contained bioactive compounds which are often proved to be more active than individual components. These phytochemicals may act against any level of biofilm formation via different mechanisms, such as quorum quenching (the foremost step of biofilm formation) and hence cell-to-cell signaling loss as found in our study. However, the study has some limitations such as studying the effectiveness of inhibiting biofilm formation, rather than disintegrating mature/preformed biofilms and focusing on *P. aeruginosa*, so the results may not apply to other bacterial strains or species that cause skin infections. Additionally, while the microtiter plate assay is very useful in vitro, it may not fully mimic in vivo conditions. As a result, the effectiveness of the extracts in actual biological systems may differ.

Future research prospects based on the current findings include investigating the potential synergistic effects of combining multiple plant extracts to enhance antibiofilm activity and reduce the risk of resistance development. Additionally, conducting animal studies to evaluate the efficacy and safety of the selected plant extracts in treating skin infections caused by *P. aeruginosa*, which would provide insights into their therapeutic potential in real biological systems. If promising results are obtained from in vivo studies, conducting clinical trials to assess the safety and efficacy of the extracts in human patients with skin infections would be necessary to pave the way for new treatments for skin infections.

## Materials and methods

### Preparation of plant extract

Liquorice (roots and rhizomes of *Glycyrrhiza glabra*, Fabaceae) was obtained from Agricultural Horticulture, Faculty of Agriculture, El-Azhar University, Cairo, Egypt. Carrot (roots of *Daucus carota* subsp. *sativus*, Apiaceae), Red Cabbage (leaves of *Brassica oleracea* var. *capitata* f. *rubra*, Brassicaceae) and beetroot (roots of *Beta vulgaris*, Amaranthaceae) were purchased from the Local Market in Cairo, Egypt. Turmeric was purchased from Local Plant Store. Neem (leaves of *Azadirachta indica*, Meliaceae) was purchased from El Mansoreya Road and placed in the Medicinal Plants station at the Faculty of Pharmacy, Ain Shams University, Cairo, Egypt. French Lavender (leaves of *L. dentata*, Lamiaceae) was obtained from Agricultural Horticulture, Faculty of Agriculture, El-Azhar University, Cairo, Egypt. Voucher specimens (PHG-P-GG-453, PHG-P-DC-454, PHG-P-BO-455, PHG-P-BV-456, PHG-P-CL-458, PHG-P-AI-457 and PHG-P-LD-459 respectively) were kept at the Pharmacognosy Department Herbarium, Faculty of Pharmacy, Ain Shams University, Cairo, Egypt. The plant names were checked with http://www.theplantlist.org in May 2024.

The collected plant parts were air-dried in the shade and cut into very small pieces/grated/sliced/crushed using hands followed by mortar and pestle/crushed. (150 g) of each plant were macerated at room temperature in (600 mL) of distilled analytical grade methanol (El Nasr Pharmaceuticals Chemicals Company (ADWIC), Egypt, PioChem for laboratory chemicals, Egypt and Lab Chem, USA) till complete exhaustion, and then the extracts were evaporated using rotavapor (BUCHI R-300, Switzerland) at 45°C using pump followed by concentrating through placing them in the hood (Flores Valles, Spain). Each plant extract was prepared in dimethyl sulfoxide (DMSO)-*d*6 (Cambridge Isotope Laboratories, Inc. Company, USA) ^45,46^ in a concentration of 10 mg/mL_DMSO_ in Eppendorf’s tubes and kept in the refrigerator until further use.

### Preparation of the reference drug

ACC (Mash Premiere company, Egypt) was prepared in a concentration of 10 mg/mL_DMSO_ in Eppendorf’s tubes and kept in the refrigerator. ACC is used as a reference compound; as it is considered a non-antibiotic drug with good antibacterial properties against *P. aeruginosa*^7,47,49^ and it is a biofilms-disrupter that showed the ability to interfere with biofilm formation and is the well-known preventer of the biofilm attachment^47^.

### Preparation of the bacterial strain and its growth

*P. aeruginosa* can infect both the skin and the urinary tract, but the way it causes these infections may differ, however its virulence factor in the two types of infection is the same which is the biofilm formation which is one of the most important virulence determinants^50^, which helps it adhere to the skin or wounds and resist antibiotic treatment. Similarly to skin infections, *P. aeruginosa* can adhere to the urinary tract lining in case of urinary tract infection and form biofilms. A multiple drug-resistant *P. aeruginosa* clinical isolate (tested against *P. aeruginosa* ATCC^®^ 27853 reference strain^51^) was recovered from the urine of patients suffering from urinary tract infection by the Department of Microbiology & Immunology, Faculty of Pharmacy, Ain Shams University and stored frozen in glycerol at -80 °C. It exhibits the highest resistance rates to Ciprofloxacin (100%), Levofloxacin (100%), Metropenem (94.7%), Ceftazidime (94.7%), Imipenem (89.5%), Gentamicin (89.5%) and Cefepime (78.9%) and lower rates of resistance to Amikacin (47.4%) and Doripenem (42.1%)^51^.

**Fig. 9:**
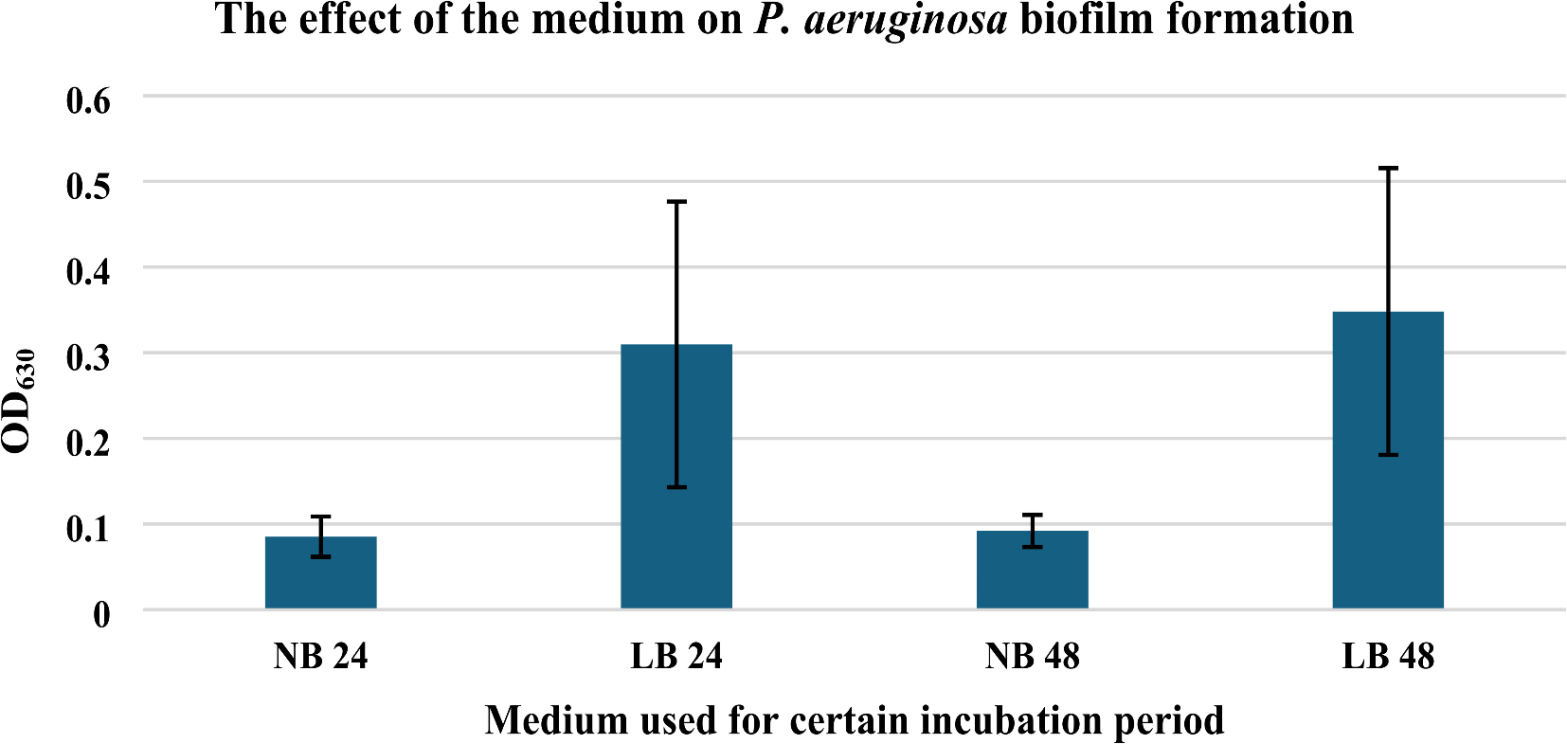
The effect of the medium on *P. aeruginosa* biofilm formation (NB: Nutrient Broth Medium, LB: Luria-Bertani broth medium supplemented with 0.1% glucose, 24: 24 h incubation period, 48: 48 h incubation period).

Different media are suitable for *P. aeruginosa* growth, such as nutrient broth, tryptone soya broth, Luria-Bertani broth, Luria-Bertani broth supplemented with 0.1% glucose and others^52^. Two media were preliminary tested which were nutrient broth and Luria-Bertani broth supplemented with 0.1% glucose. Glucose was found to efficiently promote *P. aeruginosa* biofilm formation by upregulating the expression of the extracellular polysaccharide-related gene *pslA*^53^. In case of nutrient broth medium, it was observed that the optical density (OD) of the bacterial sub-culture at 630 nm is below the cut-off value OD_630_. The cut-off OD is defined as three standard deviations above the mean OD of the negative control. In contrast, the OD_630_ in case of Luria-Bertani Broth medium supplemented with 0.1% glucose is above the cut-off OD_630_, which means that Luria-Bertani Broth supplemented with 0.1% glucose may be better used as shown in Fig. 9.

Aseptically, (1 loopful) was cultured in a test tube containing (5 mL) of autoclaved (HICLAVE HVA-110 - HIRAYAMA Manufacturing Corporation, Japan) nutrient broth ‘E’ medium (LabM, United Kingdom), incubated at 37 °C in incubator shaker (New Brunswick Scientific C25KC Classic Series - EDISON, NJ, USA) for 24 h, then (250 *µ*L) from this culture were sub-cultured in a flask containing (25 mL) Luria-Bertani broth medium (TM MEDIA, Delhi, India) supplemented with 0.1% glucose (ADWIC), incubated at 37 °C in incubator shaker for 18 h.

### Evaluation of antimicrobial activity of methanol extract of the seven plants against *P. aeruginosa*

Since antibiofilm activity is usually assessed sub-MIC values^54^ in order to state that the observed antibiofilm potential is not due to the ability of the extract to kill bacteria before biofilm formation, the antibacterial activity of the methanol extract of the seven plants against *P. aeruginosa* needs to be evaluated. The microplate method provided a potentially useful technique for determining the MICs of large numbers of natural product samples, requiring small amounts of substances. This method is not expensive and presents reproducible results^55^.

Aseptically, double-strength Luria-Bertani broth medium supplemented with 0.1% glucose (100 *µ*L) was added into 96-well flat-bottomed sterile polystyrene microtiter plate wells. (100 *µ*L) of prepared plant extract/reference were vortexed (XH-II, ARI Medical Technology Co., Ltd, China), then added to the wells, mixed well, followed by two-fold serial dilution five times, then (100 *µ*L) were discarded; to keep fixed final volume (100 *µ*L) in all wells. Bacterial suspensions (10 *µ*L of adjusted approximately equal to 10^8^ CFU/mL) were then added to these wells. Uninoculated wells are considered negative control. Microplates were incubated statically at 37 °C for 24 h. The MIC was recorded as the highest dilution showing no visible growth.

### Microtiter plate assay to evaluate the antibiofilm activity of methanol extract of the seven plants

*P. aeruginosa* was specifically chosen as it is a well-known biofilm producer and is considered the most common biofilm model organism, in addition to its relative ease of establishing biofilms formed by it in vitro. The ability of the plant extracts under investigation to reduce the biofilm formation by *P. aeruginosa* was assessed using microtiter plate assay^56^. Aseptically, (100 *µ*L) of double-strength Luria-Bertani broth medium supplemented with 0.1% glucose was added into 96-well flat-bottomed sterile polystyrene microtiter plate wells. (100 *µ*L) of prepared plant extracts were vortexed, then added to the wells, mixed well, followed by two-fold serial dilution five times. Bacterial suspensions (10 *µ*L of adjusted approximately equal to 10^8^ CFU/mL) were then added to these wells. The control used was composed of (10 *µ*L) of bacterial sub-culture on (100 *µ*L) Luria-Bertani broth medium supplemented with 0.1% glucose. The positive control used was composed of (100 *µ*L) of prepared concentration of ACC added to (100 *µ*L) of double-strength Luria-Bertani broth medium supplemented with 0.1% glucose, mixed well, followed by two-fold serial dilution five times. To exclude the DMSO effect^57^, DMSO was added to other media-contained wells (DMSO: Media: 1:1, 1:2, 1:4, 1:8, 1:16, and 1:32), then (10 *µ*L) of bacterial suspensions were added to these wells; in order to achieve the same concentrations of DMSO used in preparing plant extracts for the assay. Microplates were incubated statically at 37 °C for 24 h and for 48 h.

Planktonic cells in wells of microplates were discharged by washing three times with distilled water and shaking out water. Wells were then left to dry. Biofilms formed on the walls of microplate wells, from sessile isolates, were stained with (100 *µ*L) of filtered crystal violet (0.1% w/v) for 10 min. Crystal violet, a common dye that has been used to quantitatively assess biofilms, binds to proteins and DNA of viable cells, and thus attached cells are stained with this dye and allows visualization of the adherent biomass^58^. Crystal violet-stained wells of microplates were washed three times with distilled water, then wells were dried and blotted on a stack of paper towels to rid the plate of all excess cells and crystal violet. Acetic acid (ADWIC) (100 *µ*L of 30%) was added to each well of the microtiter plates to solubilize the crystal violet. Microtiter plates were incubated at room temperature for 15 min, then (100 *µ*L) of the solubilized crystal violet was transferred to new flat-bottomed polystyrene microtiter plates. The microplates were then measured spectrophotometrically using a plate reader (ELx808 - BioTek Instruments, Inc., USA) at 630 nm. This study was done in triplicates. Readings were processed using Gen5 Reader Control software and calculations were performed using Microsoft Excel 2016 software, Microsoft, Washington, DC, USA.

The mean % reduction of biofilm formation was determined for each plant extract at a certain concentration against *P. aeruginosa* after both 24 h and 48 h incubation period, in triplicates, using the equation below:

$$\:\text{P}\text{e}\text{r}\text{c}\text{e}\text{n}\text{t}\text{a}\text{g}\text{e}\:\left(\text{\%}\right)\text{r}\text{e}\text{d}\text{u}\text{c}\text{t}\text{i}\text{o}\text{n}=\:\left[\text{O}\text{D}\right(\text{c}\text{o}\text{n}\text{t}\text{r}\text{o}\text{l})+\text{O}\text{D}(\text{D}\text{M}\text{S}\text{O}\left)\right]-\text{O}\text{D}\left(\text{p}\text{l}\text{a}\text{n}\text{t}\:\text{e}\text{x}\text{r}\text{a}\text{c}\text{t}\right)/\text{O}\text{D}\left(\text{c}\text{o}\text{n}\text{t}\text{r}\text{o}\text{l}\right)\:\text{x}\:100\:\:$$

OD (control): OD of control wells (bacterial sub-culture on Luria-Bertani broth medium supplemented with 0.1% glucose); OD (DMSO): OD of DMSO wells at a specific concentration (bacterial sub-culture on DMSO at a specific concentration and Luria-Bertani broth medium supplemented with 0.1% glucose); OD (plant extract): OD of plant extract/reference wells at specific concentration (bacterial sub-culture on plant extract/reference at specific concentration and Luria-Bertani broth medium supplemented with 0.1% glucose).

### Statistical analysis of the results of % reduction of biofilm formation of methanol extract of the seven plants

A Two-way ANOVA was performed to examine the effects of plant extract/reference and concentration (5, 2.5, 1.25, 0.625, 0.3125, 0.15625 mg/mL) on % reduction of biofilm formation using a total sample size (the product of the number of replicating the study, the number of plant extract/reference and the number of different used concentrations) of 144 (3 × 8 × 6) in case of both 24 h and 48 h incubation periods. These analyses aimed to determine whether or not there were significant main effects of two factors: extract type (factor 1) and concentration (factor 2), as well as any interactions between these factors.

### Chemometric analysis of the % reduction of biofilm formation of methanol extract of the seven plants

Chemometric analysis was performed via PCA^59^ using Unscrambler^®^ 9.7, CAMO SA, Oslo, Norway software. Cross-validation method was utilized, and the number of PCs was adjusted to 4. PCA score plot was constructed using the % reduction of biofilm formation using the six concentrations used for the seven plant extracts under investigation after 24 h and 48 h incubation periods.

### Chemical investigation of methanol extract of *L. dentata* leaves using LC-MS analysis

The hyphenated high performance liquid chromatography-mass spectrometry (HPLC-MS) technique is an important method used for identifying complex mixtures especially the phenolics in plant extract, by comparing the mass spectrum obtained with literature (tentative identification)^13,60^. LC-MS analysis was performed on methanol extract of *L. dentata* leaves which showed the highest bioactivity. LC-MS analysis was performed using HPLC (Nexera LC-30AD) equipped with an autosampler (SIL-30AC), temperature-controlled column oven (CTO-20AC), and coupled to triple quadrupole mass spectrometer (Nexera with LCMS-8045, Shimadzu Corporation, Kyoto, Japan) that was equipped with reversed-phase (RP)-C18 UPLC column (shimpack 2 mm × 150 mm) possessing 2.7 *µ*m particle size. The following gradient elution, using HPLC-grade acetonitrile (ACN) and water (Sigma Aldrich Company), was used (ACN, 0. 1% HCOOH in H_2_O) 0–2 min (10% ACN); 2–5 min (30% ACN-80% ACN), 5–15 min (50% ACN), 15–25 min (70% ACN), 25–28 min (80% ACN), 28–30 min (80% ACN) and 30–33 min (10% ACN), with 0.2 mL/min flow rate. Positive and negative modes were operated during LC-MS with electrospray ionization. LC-MS data were collected and processed by Lab Solutions software.

### Molecular docking analysis of the identified compounds of methanol extract of *L. dentata* leaves

Since *P. aeruginosa* is the most studied microorganism with regard to QS^50^ which is a communication mechanism used by bacteria to regulate gene expression in response to population density, further research was needed to fully elucidate the interactions between *L. dentata*-derived compounds and QS systems in *P. aeruginosa*. Molecular docking analysis was done against LasR, the QS regulatory protein, of *P. aeruginosa*. Molecular docking analysis was done against LasR using BIOVIA Discovery Studio 2016 Client. The most common approximation for docking is to hold the protein in a rigid conformation and dock a series of ligand conformations into the active site. Fast docking based on binding site features (“hotspots”) was done using LibDock which is an algorithm for docking small molecules into an active receptor site. Initially, a hotspot map is calculated for the receptor active site which contains polar and apolar groups. This hotspot map is subsequently used to rigidly align the ligand conformations to form favorable interactions. After a final energy-minimization step (allowing the ligand poses to be flexible), the top-scoring ligand poses are saved.

Protein structure was downloaded from https://www.rcsb.org/structure/2UV0. Water was removed from the downloaded protein, then the protein was cleaned to add hydrogens, check bonds and bond orders, and correct them, if necessary, standardize atom order in amino acids, and modify terminal residues. The force field is then applied. Fixed atom constraints were created from atoms rather than hydrogens. The prepared protein was then defined as a receptor and the binding site sphere was defined. Ligands (identified compounds from methanol extract of *L. dentata* leaves along with ACC) were subjected to adding hydrogens (if absent), optimizing their geometries using a fast, Dreiding-like forcefield, then were applied to force field using CHARMm which has a wide coverage for general organic molecules.

Ligands were then prepared to fix bad valencies, generate 3D coordinates, and remove duplicate structures. LibDock docking program performs the following steps using a set of pre-generated ligand conformations and a receptor with a specified binding site: removing hydrogen atoms, ranking ligand conformations and pruning by solvent-accessible solvent area, finding hotspots using a grid placed into the binding site and using polar and apolar probes. The number of hotspots was pruned by clustering to 200. Docking ligands pose was done by aligning to the hotspots. This was performed by using triplets (i.e., three ligand atoms are aligned to three receptor hotspots). Poses that result in protein clashes are removed. A final Broyden-Fletcher-Goldfarb-Shanno pose optimization stage is performed using a simple pair-wise score (similar to Piecewise Linear Potential). The top-scoring ligand poses are retained, then hydrogen atoms are added.

## Conclusion

The seven tested plants (liquorice, carrot, red cabbage, beetroot, turmeric, neem, and French lavender) were selected due to their reported usage against various skin ailments. Using microtiter plate assay, the current study revealed that the methanolic extracts of all tested plants can significantly reduce the formation of *P. aeruginosa* biofilm with different degrees at both 24 h and 48 h. In particular, the methanol extract of turmeric was found to be more effective than pure curcumin activity previously reported, while the methanol extract of neem leaves was effective at lower concentrations than previously reported. This study also revealed that the methanol extracts of carrot and *L. dentata* leaves were effective against *P. aeruginosa* biofilm for the first time. *L. dentata* leaves, the superior among the seven tested plants with 0.625 mg/mL as optimum concentration, are reported for the first time herein to have anti-bacterial biofilm activity and specifically anti-*P. aeruginosa* biofilm activity. Chemometric analysis has confirmed the closeness of French lavender in its activity to the reference (ACC) used. This study is one of the few to examine *L. dentata* leaf extract rather than its essential oil. This finding suggests that *L. dentata* leaves may be economically significant and repurposed for drug discovery. Further investigations of the methanol extract of *L. dentata* leaves using LC-MS analysis identified several compounds that have not been previously reported in *L. dentata*, including coumaric acid, ferulic acid, fertaric acid and chicoric acid. The major identified compound, sagerinic acid, was found to be responsible for the antibiofilm activity of *L. dentata* extract against LasR, one of QS regulatory proteins of *P. aeruginosa* using molecular docking study. By integrating traditional knowledge with modern scientific methodologies, this study concludes that *L. dentata* leaves have therapeutic potential against biofilm-associated skin infections, paving the way for the development of innovative antimicrobial agents. Further research is recommended to be carried out to fully elucidate the therapeutic properties and mechanisms of action of *L. dentata* leaves as a new valuable botanical resource in medicine and healthcare.

## Data Availability

Data supporting reported results can be provided upon request via e-mail: maram.aboulwafa@pharma.asu.edu.eg. For more information about data citations, you can find them at the “References” section.
